# Tandem light-emitting technology accelerates the commercial application of perovskite LEDs

**DOI:** 10.1038/s41377-024-01624-w

**Published:** 2024-10-16

**Authors:** Xiang Zhang, Jiajun Luo, Enguo Chen, Abd. Rashid bin Mohd Yusoff

**Affiliations:** 1 Wuhan National Laboratory for Optoelectronics and School of Optical and Electronic Information, Huazhong University of Science and Technology, Wuhan, 430074 Hubei China; 2 National and Local United Engineering Laboratory of Flat Panel Display Technology, College of Physics and Information Engineering, Fuzhou University, Fuzhou, Fujian 350108 China; 3 Department of Physics, Faculty of Science, Universiti Teknologi Malaysia, Johor Bahru, Malaysia

**Keywords:** Inorganic LEDs, Photonic devices

## Abstract

Hybrid tandem perovskite-organic LED has been developed to achieve high external quantum efficiency, narrow linewidth, and extended device lifespan, which shows great promise for future perovskite-EL-based commercial applications.

Perovskite light-emitting diodes (PeLEDs) with advantages in their superior color quality and cost-effectiveness are reaching organic LEDs (OLEDs)’ performance and currently emerging as potential contenders^1–5^. Despite the fast advances in the electroluminescence (EL) performance of PeLEDs, their stability still lags behind state-of-the-art OLEDs due to the ion migration driven by electric field. Integrating emerging narrow-band emitters with mature display technologies such as liquid crystal display (LCD) and OLEDs to expand the display color gamut could provide a viable pathway to expedite commercialization. For example, LCD TVs equipped with quantum dots (QDs) backlights had already been commercialized in 2021. Samsung has produced commercial OLED TVs with QDs color conversion. Similarly, perovskite QD films have also been produced as backlights in LCD TVs^6^. As a potential commodity market application, there is no doubt that these emerging technologies in photoluminescent (PL) application scenarios bring exciting prospects to the display market. More importantly, it also highlights the significance of advancing the perovskite-EL displays.

A tandem structure can effectively merge various types of light-emitting devices together by stacking two or more LED units through a charge generation layer (CGL), which has been extensively employed in OLEDs for practical display applications. Such a tandem structure offers notable advantages. Firstly, it enables higher external quantum efficiency (EQE), nearly the combined efficiencies of each light-emitting unit. Secondly, the device lifespan is extended as the tandem device requires less current than a single device to achieve equivalent brightness.

In a newly published paper in *Light: Science & Applications*, Xuyong Yang et al. from Shanghai University and Kai Wang et al. from Southern University of Science and Technology have proposed a hybrid tandem device structure by combining a bottom PeLED and a top OLED^7^. This distinctive tandem device structure magnifies the strengths of the two types of display technologies and enables complementary performance. Specifically, PeLED and OLED with consistent emission peaks are selected to maximize photon emission and achieve narrow EL spectra of tandem LEDs by leveraging the narrow-band emission characteristics of perovskite materials. It has been found that incorporating an ultrathin MoO_3_ layer into HAT-CN/CBP overcomes the trade-off between optical and electrical properties of the CGL structure, and significantly reduces Joule heating of the tandem LEDs under operation. Therefore, the efficient CGL enables the tandem LED to achieve a high EQE of over 40%, high color purity (narrow full-width at half-maximum of ~30 nm), and excellent operational half-lifetime of over 40,000 h.

Since combining two different types of LED is actually different from conventional tandem OLEDs/QLEDs^8–10^, it requires comprehensive understanding including the design of an efficient CGL for a lossless electrical connection, optimization of a balanced charge transport in each unit with different electrical characteristics, and control over an ideal position for optical interference. The aforementioned discoveries are not only encouraging but also indicate the potential for further advancements in display technology using a similar device architecture. This provides an important guideline for integrating different types of light-emitting devices into practical display applications. It is foreseeable that a tandem structure incorporating a PeLED with an OLED can realize a vivid and efficient display, capitalizing on the well-established manufacturing processes of OLEDs.
